# Modified coronally advanced tunnel technique with enamel matrix derivative in addition to subepithelial connective tissue graft compared with connective tissue graft alone for the treatment of multiple gingival recessions: prognostic parameters for clinical treatment outcomes

**DOI:** 10.1007/s00784-021-04045-w

**Published:** 2021-07-04

**Authors:** Bartłomiej Górski, Renata Górska, Marcin Szerszeń, Tomasz Kaczyński

**Affiliations:** 1 Department of Periodontology and Oral Mucosa Diseases, Medical University of Warsaw, Stanisława Binieckiego St 6, 02-097 Warsaw, Poland; 2 Department of Dental Prosthetics, Medical University of Warsaw, Stanisława Binieckiego St 6, 02-097 Warsaw, Poland

**Keywords:** Enamel matrix derivative, Esthetics, Logistic regression, Modified coronally advanced tunnel technique, Multiple gingival recessions

## Abstract

**Objectives:**

To investigate factors that influence 12-month outcomes after treatment of multiple gingival recessions (GR) with modified coronally advanced tunnel (MCAT) and subepithelial connective tissue graft (SCTG) with enamel matrix derivative (EMD) (tests) or without (controls).

**Materials and methods:**

Twenty patients with 150 GR were treated. Logistic regression models were used to identify baseline parameters that could predict 12-month average root coverage (ARC), complete root coverage (CRC), root esthetic coverage score (RES), gain in keratinized tissue width (KTW), and gain in gingival thickness (GT).

**Results:**

The likelihood of ARC > 85% increased sevenfold (odds ratio [OR] = 7.33; 95% confidence interval [CI] = 2.43–12.12), of achieving CRC: 21-fold (OR = 21.23; 95% CI = 10.21–45.32), and of gaining RES = 10: tenfold (OR = 10.23; 95% CI = 5.78–32.23) in favor of EMD-treated sites. With each 1-mm^2^ increase in baseline avascular exposed root surface area (AERSA), the odds of failure (ARC ≤ 85%, not achieving CRC and postoperative GT ≤ 2 mm) increased almost fourfold (OR = 3.56; 95% CI = 1.98–10.19), fourfold (OR = 4.23; 95% CI = 1.11–9.02), and nearly sixfold (OR = 5.76; 95% CI = 2.43–12.87), respectively. The greater the baseline GT, the more likely the chance of achieving CRC (OR = 10.23; 95% CI = 8.37–16.23) and RES = 10 (OR = 5.50; 95% CI = 3.34–16.43). All models exhibited fair to excellent discrimination and satisfactory calibration.

**Conclusions:**

Based on logistic regression, EMD application improved postoperative ARC, CRC and RES; baseline AERSA predicted 12-month ARC, CRC, and GT gain, whereas baseline GT was a predictor of achieving CRC and perfect RES.

**Clinical relevance:**

Additional use of EMD, lower baseline AERSA, and greater baseline GT significantly increase the odds of obtaining better outcomes 12 months after MCAT + SCTG technique.

## Introduction

Gingival recession (GR) is the displacement of gingival margin apical to the cemento-enamel junction (CEJ) which contributes to the exposure of root surface [1]. These defects were categorized following the 2018 World Workshop into three categories: (1) recession type 1 (RT1) with no loss of interproximal attachment, (2) recession type 2 (RT2) when the amount of interproximal attachment loss is lower than of buccal attachment loss, and (3) recession type 3 (RT3) if interproximal attachment loss is greater than buccal attachment loss [2]. The prevalence of GR was found in 91.6% of patients and it decreased to 70.7% when only esthetic zone was considered [3]. The whole-mouth patient-level prevalence of RT1, RT2, and RT3 was 12.4%, 88.8%, and 55.0%, respectively. Several predisposing factors for the occurrence of GR were suggested, such as thin periodontal phenotype, improper toothbrushing, the presence of restorations with intra-crevicular margins, orthodontic treatment, and persistent gingival inflammation. If left untreated, the progression of buccal GR equaled 0.4 mm over an average follow-up of 4 years [4].

Treatment for GR typically results in esthetic improvement, elimination of dentin hypersensitivity, and minimized risk of root caries. Available data from recent literature indicate that tunnel technique (TUN) is a highly effective and predictable procedure in the treatment of multiple GR defects. According to current systematic review and meta-analysis, the overall calculated average root coverage (ARC) of tunnel for multiple GR was 87.87 ± 16.45%, whereas complete root coverage (CRC) could be achieved in 57.46% of defects [5]. In another network meta-analysis, Cairo et al. [6] stated that TUN + SCTG technique was significantly associated with higher root coverage esthetic score (RES) than coronally advanced flap (CAF) (0.84 [95% CI = 0.15–1.53]; p = 0.01). However, no significant difference between TUN + SCTG and CAF + SCTG was detected (0.09 [95% CI =  − 0.54–0.72]; p = 0.77). The authors concluded that graft material might have a bigger impact on esthetic outcomes than the flap design.

Positive outcomes of root coverage with TUN might be attributed to inherent advantage of this approach being a minimally invasive procedure with limited flap opening and lack of vertical releasing incisions, all of which contribute to decreased tissue trauma, enhanced wound healing, and greater blood supply to the graft. The tunnel approach was first described by Zabalegui et al. as a split flap preparation of buccal tissues [7]. The use of subepithelial connective tissue graft (SCTG) further improved clinical outcomes [8]. Over the following years, additional modifications of this technique were proposed, such as introduction of the microsurgical approach [9], full-thickness flap preparation, papillae detachment and elevation, modified suturing technique [10, 11], application of collagen porcine dermal matrix in lieu of SCTG [12], inclusion of biological factors such as enamel matrix derivative (EMD) [13] or concentrated growth factor (CGF) [14], and site-specific application of SCTG [15]. A clinical benefit for the addition of EMD to MCAT has been found in some studies [16, 17], while others did not observe any difference between the treatment modalities [10, 13, 18].

Considerable evidence has pinpointed possible factors potentially associated with clinical outcomes after surgical treatment of GR. Such factors include patient (plaque control, smoking, general health, compliance), preoperative site-specific characteristics (recession depth and width, presence of keratinized tissue, gingival thickness and type of phenotype, loss of interproximal attachment, tooth type and tooth location, presence of frenuli), and surgical procedures (flap design, root surface biomodification, type of graft, flap tension), to name a few [8, 19–24]. However, the majority of data were presented with descriptive statistics that were used to analyze changes in evaluated parameters during follow-up periods. To predict outcomes of implemented treatment and to adjust for confounding factors, logistic regression models are often created [25]. This method utilizes clinical characteristics (predictors or independent variables) to estimate the likelihood of a specific outcome (dependent variable). Logistic regression determines which of the assessed factors have the strongest relation with chosen end points with the least variable [25]. A perfect model should not only discriminate well a particular outcome but also be satisfactorily calibrated to assign correct actual chance. In case of root coverage, regardless of the magnitude of ARC, average KTW, or GT gain, clinicians might be interested in using models that perform well on specific endpoints, such as postoperative CRC or perfect RES (10), which remain pivotal goals for both the patient and clinicians. It is commonly understood that precision medicine calls for personalized diagnosis and treatment.

The purpose of the prior report was to investigate the adjunctive use of EMD to MCAT + SCTG in the treatment of multiple RT1 and RT2 [17]. The primary outcomes of the study were ARC and CRC, while the secondary outcomes included changes in GR height, recession width (RW), clinical attachment level (CAL), gingival thickness (GT), keratinized tissue width (KTW), RES, and patient-reported outcomes. However, to the best of the authors’ knowledge, no studies have yet evaluated possible baseline parameters that could predict specific outcomes after root coverage of multiple RT1 and RT2 with MCAT using logistic regression analysis. Therefore, this article aims exclusively to (1) identify potential predictors of ARC, CRC, RES, KTW gain, or GT gain 12 months after treatment of multiple GR with MCAT, SCTG, and EMD; (2) calculate odds ratios (OR) and their associated 95% confidence intervals (CI) to measure how strong the relations between different variables and designated outcomes are; and (3) investigate the validity of developed models by evaluation of discrimination and calibration. Observation of feasible preoperative factors might help clinicians predetermine the likelihood of achievable outcomes after MCAT and in the decision-making process for multiple GR treatment, all of which is of considerable clinical relevance.

## Materials and methods

### Study design and subject population

**Fig. 1:**
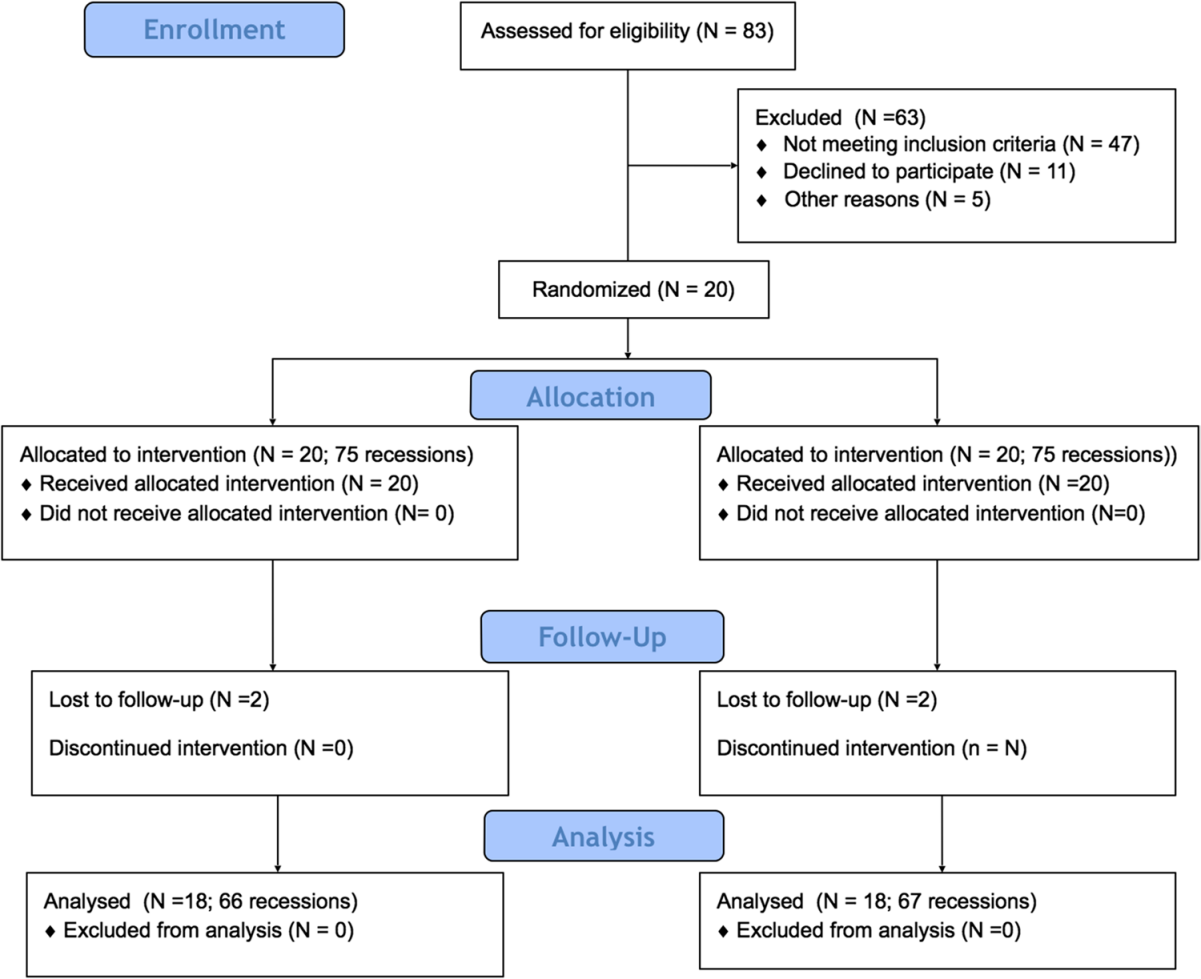
Consort diagram showing the study design

This study was based on extended material of a previously reported randomized clinical trial [17]. The research was performed in accordance with the Helsinki Declaration of 1975, as revised in Tokyo in 2004 after getting positive approval of the Bioethics Committee of Medical University of Warsaw (KB/208/2017). The study protocol was registered with ClinicalTrials.gov (registration number: NCT03354104). The subject population was recruited among patients referred to the Department of Periodontology and Oral Mucosa Diseases of Medical University of Warsaw between January 2018 and June 2019. Patients were qualified into the study by one examiner (TK). Once the selected subjects agreed to participate in the study by signing an informed consent form, they were instructed on how to use the roll technique with a soft toothbrush, and provided with dental prophylaxis and polishing. Twenty patients (13 women and 7 men, aged 21–38; mean age 28.35 ± 4.51 years) were enrolled in the study and one hundred fifty gingival recessions (131 RT1 and 19 RT2) were treated in the split-mouth manner (one side of the maxilla or mandible served as test and the opposite side as control). The defects were treated with MCAT in combination with SCTG either with (test, 75 defects) or without EMD (control, 75 defects). All clinical parameters and outcomes were assessed at baseline, after 6 months, and for the present study, after 12 months (Fig. 1).

With the assumption that percentage of root coverage was the primary objective, and based on the data that standard deviation (SD) of the differences in the paired measurements would not surpass 30%, the sample size was determined to be 18 subjects per treatment group for paired continuous data [26]. This would provide 80% power to disclose a true difference of 20% points between test and control. Keeping in mind that some patients could be lost during follow-up, 20 patients were enrolled in the study.

Clinical parameters were assessed by a calibrated examiner (SW), who was blinded to the surgical procedures. For the calibration exercise, six non-study patients with at least two contralateral teeth with recessions were recruited and subsequently evaluated with an interval of 24 h between recordings. Calibration was accepted when ≥ 90% of the recordings were reproduced within a difference of 1.0 mm and an exact agreement could be repeated in 75% of measurements.

### Inclusion and exclusion criteria

The inclusion criteria were as follows: (1) at least two adjacent gingival recessions of recession type I and/or II at least 1 mm deep at homologous teeth in maxilla or mandible; (2) full-mouth plaque score (FMPS) < 20%; (3) full-mouth bleeding on probing (FMBOP) < 20%; (4) presence of identifiable cemento-enamel junction (CEJ); and (5) over 18 years of age. The exclusion criteria were as follows: (1) active periodontal disease; (2) caries lesions or restorations in the cervical area; (3) systemic diseases with compromised healing potential or infectious disease; (4) use of medications affecting periodontal status; (5) smoking; and (11) pregnancy or lactation.

### Clinical measurements

The following parameters were measured using a graded periodontal probe (UNC probe 15 mm, Hu-Friedy) at the mid-aspect of the involved teeth under local anesthesia: (1) gingival recession height (GR), distance from CEJ to gingival margin; (2) RW, distance measured horizontally between the bilateral gingival margins at CEJ level; (3) probing pocket depth (PPD), distance from gingival margin to the bottom of gingival sulcus; (4) CAL, distance from CEJ to the bottom of gingival sulcus; (5) KTW, distance from gingival margin to muco-gingival junction (MGJ); MGJ was demarcated by staining the muco-gingival complex with iodine solution (Lugol’s solution); (6) GT, measured 3 mm apically from gingival margin with the use of endodontic spreader 25 ISO (Poldent, Warsaw, Poland) and a silicon stopper put perpendicularly to the gingival surface until alveolar bone or root surface was reached, and an electronic caliper (YATO YT-7201, Toya, Wrocław, Poland) with 0.01 mm accuracy was selected to calculate GT value; (7) FMPS, the percentage of total surfaces (four aspects per tooth: buccal, lingual, mesial, distal) that revealed presence of plaque [27]; (8) FMBOP, the percentage of total points (four points per tooth: mesio-buccal, mid-buccal, disto-buccal, mid-lingual) that bled after gentle probing [28]. GR, RW, PPD, CAL, KTW, and GT were recorded to the nearest 0.5 mm at baseline and 12 months after surgery and the percentage of root coverage was measured.

### Randomization and allocation concealment

Randomization was performed before surgical treatment by a statistician who was not involved in the study by means of a computerized random number generator. Allocation of treatment sites was concealed in sealed and opaque envelopes. It was revealed to the surgeon just before the procedure. One envelope was opened to designate the surgical site located to the right to one of the two treatment protocols; subsequently, the surgical site to the left was treated in accordance with opposite modality. Patients were not informed on the allocation.

### Surgical treatment

The surgical procedures were performed in accordance with modified coronally advanced tunnel technique by one surgeon (BG) [9]. Both sides were treated during the same visit. After local anesthesia with 4% articaine hydrochloride with adrenaline (1:100,000) (Ubistesin Forte 1.7 mL, 3 M ESPE, Saint Paul, MN, USA), a full-thickness flap with up to MGJ was prepared with a small elevator and above MGJ a split-thickness flap was raised using tunneling instruments. The papillary regions were detached in their buccal aspects with the periosteum. The exposed roots were planed using designated curettes. Then, SCTG was harvested from the palate as epithelialized gingival graft [29]. After removing epithelium, the thickness of SCTG was less than 1 mm, and its width was around 4 mm. A hemostatic sponge was placed stabilized in the donor area with cross mattress non-resorbable sutures (Seralon 4/0 18 mm 3/8, Serag-Wiessner GmbH & Co. KG, Naila, Germany). The exposed roots were soaked with 24% EDTA (PrefGel, Straumann, Basel, Switzerland) for 2 min, washed with saline and subsequently conditioned with EMD (Emdogain®, Straumann). SCTG was inserted inside the tunnel and stabilized at CEJ or 1 mm below the CEJ with resorbable sling sutures (PGA Resorba 6/0 11 mm 3/8, RESORBA Medical GmbH, Nürnberg, Germany). In the next step, the mobilized buccal flap was advanced coronally to fully cover SCTG, and secured with 6/0 non-resorbable monofilament sling sutures (Seralon 6/0 12 mm 3/8, Serag-Wiessner GmbH & Co). On the control site, the recipient area was treated similarly to those of the test group, but neither 24% EDTA nor EMD was used.

### Postoperative instructions and evaluation of morbidity

The subjects took 400 mg of ibuprofen after surgery and were asked to take the second dose after 8 h. Any additional tablets were taken later if necessary. The patients were informed to avoid brushing, flossing, and chewing in the treated area for the period of 2 weeks and to rinse the mouth twice daily for 1 min using 0.2% chlorhexidine solution. Sutures were removed after 14 days and the patients were instructed in mechanical tooth cleaning of the operated sides using a soft toothbrush and the roll technique. Check-up appointments were scheduled at 1, 3, 6, and 12 months for professional oral hygiene procedures and control.

### Evaluation of esthetics

The esthetic outcome was assessed 12 months postoperatively by an independent examiner (TK), who was blinded to the treatment allocation. Five variables were analyzed on comparing digital photographs taken at baseline and after 12 months: (1) gingival margin (GM): 0 points in case of failure of root coverage, 3 points in case of partial root coverage, and 6 points in case of complete root coverage (CRC); (2) marginal tissue contour (MTC): 0 points in case of irregular gingival margin, and 1 point in case of ideal marginal contour; (3) soft tissue texture (STT): 0 points in case of scar formation, and 1 point in case of absence of scar; (4) muco-gingival junction alignment: 0 points in case of MGJ not aligned with the MGJ of adjacent teeth, and 1 point in case of MGJ aligned with the MGJ of adjacent teeth; (5) gingival color (GC): 0 points when color of tissue varies from gingival color at adjacent teeth, and 1 point in case of proper color [30]. The highest and best esthetic score to be achieved was 10.

### Statistical analysis

Statistical analysis was carried out with R 3.2.3 software (R Core Team 2019). The significance level of 0.05 was adopted. The clinical and esthetic parameters were compared between the two groups. Descriptive statistics were performed using mean values, standard deviations (SD), frequencies, and percentages. Normality of distribution for quantitative variables was assessed by means of the Shapiro–Wilk test. Due to data normal distribution, differences between baseline and 12 months and differences between the groups at each time point were compared with the Student *t* test. Comparison of fractions (percentages) was carried out using Pearson’s chi-square test. To evaluate the outcomes of the treatment, the following calculations were made: (1) recession reduction = GR0 − GR12, (2) average root coverage (ARC) = GR0 − GR12 / GR0 × 100%, (3) CAL gain = CAL0 − CAL 12, (4) KTW gain = KTW12 − KTW0, (5) GT gain = GT12 − GT0, and (6) avascular exposed root surface area (AERSA) = GR × RW.

In an attempt to determine independent preoperative factors that were associated with 12-month clinical outcomes, five logistic regression models were set by the following dependent variables: (1) ARC (binary variable; ≤ 85% low group and > 85% high group); (2) CRC (binary variable); (3) RES (binary variable; ≤ 9 low group and 10 high group); (4) KTW gain (binary variable; ≤ 3 mm low group and > 3 mm high group); (5) GT gain (binary variable; ≤ 2 mm low group and > 2 mm high group). As independent variables, patients’ age, patients’ sex, the use of EMD, tooth type (incisors/canines/premolars/molars), tooth position (upper/lower), GR, RW, AERSA, PPD, CAL, KTW, GT, and RT at baseline were used. All of the abovementioned parameters were evaluated with multiple regression models. Final regression models were acquired using stepwise selection of predictors with backward elimination. The strict entry criteria excluded recruitment of smokers and patients with inadequate oral hygiene and high residual infection; hence, smoking, FMPS, and FMBOP values were not considered in the analysis. The results were presented as OR and 95% CI.

Receiver operating characteristic curves (ROC) were constructed, and the areas under the curve (AUC) and their 95% CI were evaluated for measuring of discrimination of each developed model with regard to chosen cutoff points for ARC, CRC, RES, KTW gain, and GT gain. A model with no better accuracy than chance would have an AUC of 0.5, a model with fair accuracy would have an AUC higher than 0.7, a model with excellent accuracy would have an AUC higher than 0.9, and a model with perfect sensitivity and specificity would have an AUC of 1 [31]. At this stage, calibration of models was analyzed to accurately estimate the absolute probability of particular outcomes. The Hosmer–Lemeshow goodness of fit (GOF) test was used to measure statistical significance of any differences between predicted and observed outcomes. The p value of 0.1 or higher indicated that the model was well calibrated, the p values of < 0.1 and > 0.5 meant that the model was neither well calibrated nor grossly miscalibrated, and the p value < 0.5 indicated miscalibrated estimates [32]. This approach was chosen for the construction of final presentations of models.

## Results

### Patient characteristics

A total of 150 gingival recessions were treated (75 defects in the SCTG + EMD group and 75 defects in the SCTG group). GR distribution characteristics are shown in Table 1. The majority of treated teeth were upper premolars. Fourteen subjects presented recessions in the maxillary arch, and the other six had defects in the mandibular arch. Contralateral test and control defects were well balanced, and baseline data were homogeneous for all of the 20 involved patients (Table 2). Healing was uneventful in all subjects, all of whom completed scheduled appointments and the 6-month follow-up. However, two patients were lost to follow-up between 6 and 12 months. Consequently, a total of 132 gingival recessions were analyzed in 18 subjects.

**Table 1:** Characteristics of the test and control groups

Variables	Baseline		12 months	
	Test (*N* = 20; *n* = 75)	Control (*N* = 20; *n* = 75))	Test (*N* = 18; *n* = 66)	Control (*N* = 18; *n* = 67)
Tooth type (*n*)				
Incisors	14	14	13	14
Canines	17	17	14	14
Premolars	35	35	31	31
Molars	9	9	8	8
Tooth position (*n*)				
Maxillary teeth	56	58	49	49
Mandibular teeth	19	17	17	18
Type of GR according to Cairo (*n*, %)				
RT1	65 (86.7)	66 (88)	60 (91)	61 (91)
RT2	10 (13.3)	9 (12)	6 (9)	6 (9)

*N* number of patients, *n* number of defects, *GR* gingival recession, *RT* recession type

**Table 2:** Clinical parameters (mean and standard deviation) at baseline and 12 months after surgery

	Baseline	12 months	*p*
GR SCTG + EMD (mm)	2.22 (1.00)	0.11 (0.35)	< 0.0001*
GR SCTG	2.16 (1.02)	0.21 (0.48)	< 0.0001*
*p*	0.7171	0.2219	
ARC SCTG + EMD (%)		95.00 (18.27)	
ARC SCTG		91.00 (23.34)	
*p*		0.5693	
GR red SCTG + EMD (mm)		2.09 (0.92)	
GR red SCTG		2.04 (1.13)	
*p*		0.4828	
RW SCTG + EMD (mm)	3.30 (1.38)	0.57 (1.66)	< 0.0001*
RW SCTG	3.25 (1.42)	0.42 (1.34)	< 0.0001*
*p*	0.7171	0.3321	
AERSA SCTG + EMD (mm^2^)	7.61 (0.43)	0.45 (0.05)	< 0.0001*
AERSA SCTG	7.74 (0.38)	0.54 (0.07)	< 0.0001*
*p*	0.0783	0.0329*	
PPD SCTG + EMD (mm)	1.44 (0.58)	1.66 (0.68)	0.4536
PPD SCTG	1.43 (0.52)	1.76 (0.71)	0.1622
*p*	0.8822	0.1901	
CAL SCTG + EMD (mm)	3.56 (1.19)	1.42 (0.86)	< 0.0001*
CAL SCTG	3.25 (1.18)	1.62 (0.98)	< 0.0001*
*p*	0.1137	0.0416*	
CAL gain SCTG + EMD (mm)		2.13 (1.05)	
CAL gain SCTG		1.62 (1.48)	
*p*		0.0120*	
KTW SCTG + EMD (mm)	2.63 (1.42)	3.34 (1.27)	0.2310
KTW SCTG	2.55 (1.27)	3.23 (1.35)	0.2511
*p*	0.3190	0.3382	
KTW gain SCTG + EMD (mm)		0.75 (1.00)	
KTW gain SCTG		0.71 (1.12)	
*p*		0.4429	
GT SCTG + EMD (mm)	1.16 (0.34)	2.05 (0.62)	< 0.0001*
GT SCTG	1.18 (0.33)	2.14 (0.79)	< 0.0001*
*p*	0.1537	0.8831	
GT gain SCTG + EMD (mm)		0.91 (0.61)	
GT gain SCTG		1.00 (0.71)	
*p*		0.6521	

*GR* gingival recession height, *SCTG* subepithelial connective tissue graft, *EMD* Emdogain®, *ARC* average root coverage, *GR red* gingival recession reduction, *RW* gingival recession width, *AERSA* avascular exposed root surface area, *PPD* probing pocket depth, *CAL* clinical attachment level, *KTW* keratinized tissue width, *GT* gingival thickness. *Statistically significant (*p* ≤ 0.05)

### Clinical outcomes

The clinical results at 12-month follow-up are depicted in Table 2. At 12 months, PPD values were not statistically different within and between groups. Significant decreases in GR, RW, and CAL were noted in both groups from baseline to 12 months compared with the baseline measurements. In the test group, the mean recession height decreased significantly from 2.2 ± 1.0 (baseline) to 0.1 ± 0.3 mm (12 months), with percentage of ARC of 95 and CRC in 60 out of 66 (90%) recession defects. In the control group, the mean recession height decreased significantly from 2.1 ± 1.0 to 0.2 ± 0.4 mm, with percentage of ARC of 91 and CRC in 58 out of 67 (86%) recession defects. The abovementioned differences between tests and controls were not statistically significant and the observed changes were perfectly comparable between groups. From baseline to 12 months, there was also a statistically significant CAL gain in the two groups (2.1 ± 1.0 and 1.6 ± 1.4 mm for the test and control groups, respectively). A significantly higher gain in CAL was found in patients treated by the SCTG + EMD technique (p = 0.0120). KTW and GT increased significantly and similarly on both sides: for KTW, from 2.6 ± 1.4 to 3.3 ± 1.2 mm on the SCTG + EMD side and from 2.5 ± 1.2 to 3.2 ± 1.3 mm on the SCTG side; for GT, from 1.1 ± 0.3 to 2.0 ± 0.6 on the SCTG + EMD side and from 1.1 ± 0.3 to 2.1 ± 0.7 on the SCTG site. No significant differences with respect to WKT and GT gain between the two treatment modalities were observed at 12 months.

### Root coverage esthetic score

**Fig. 2:**
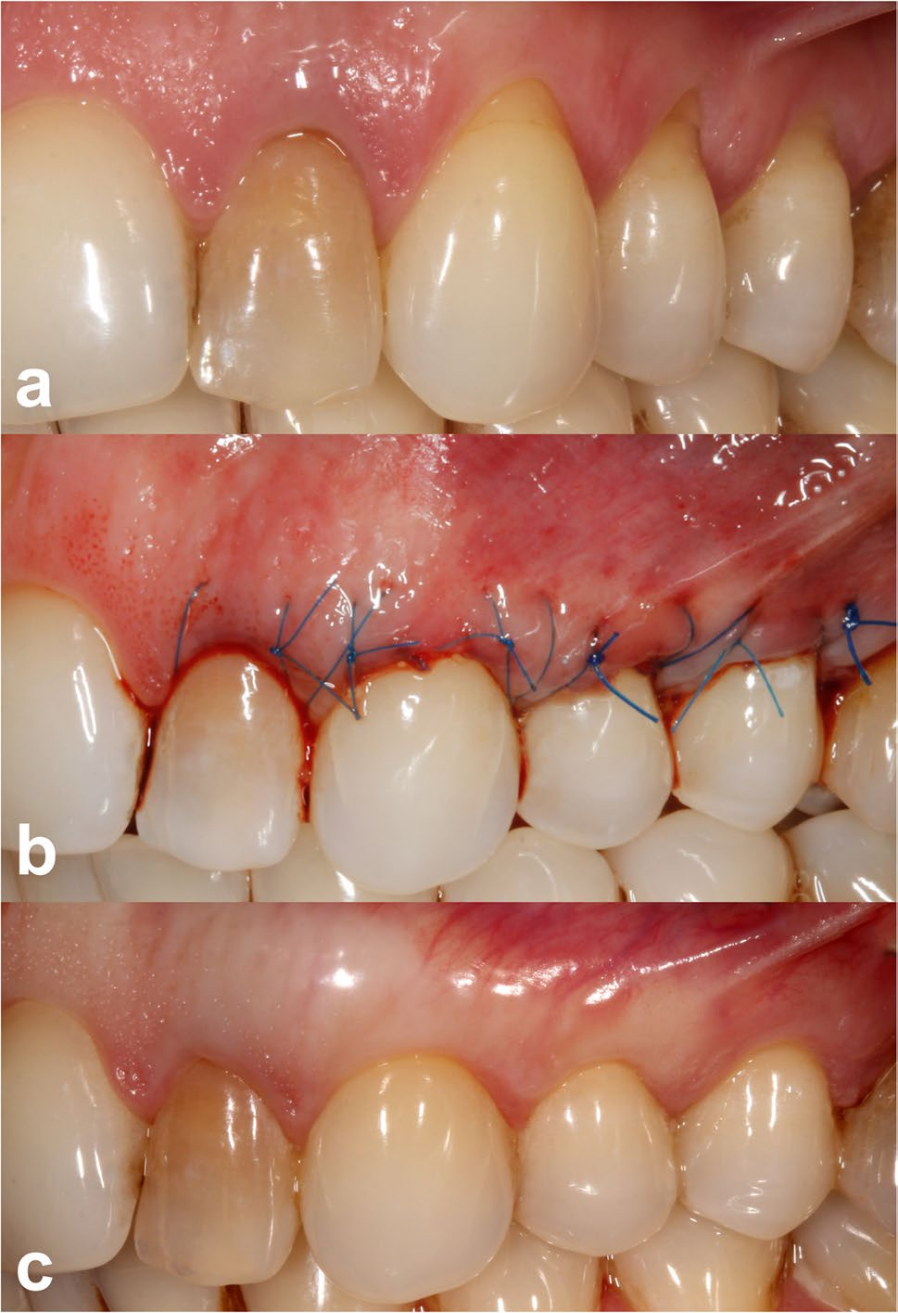
**a** Preoperative view of gingival recessions on test side. **b** Immediate postoperative view. **c** Twelve months postoperative view

**Fig. 3:**
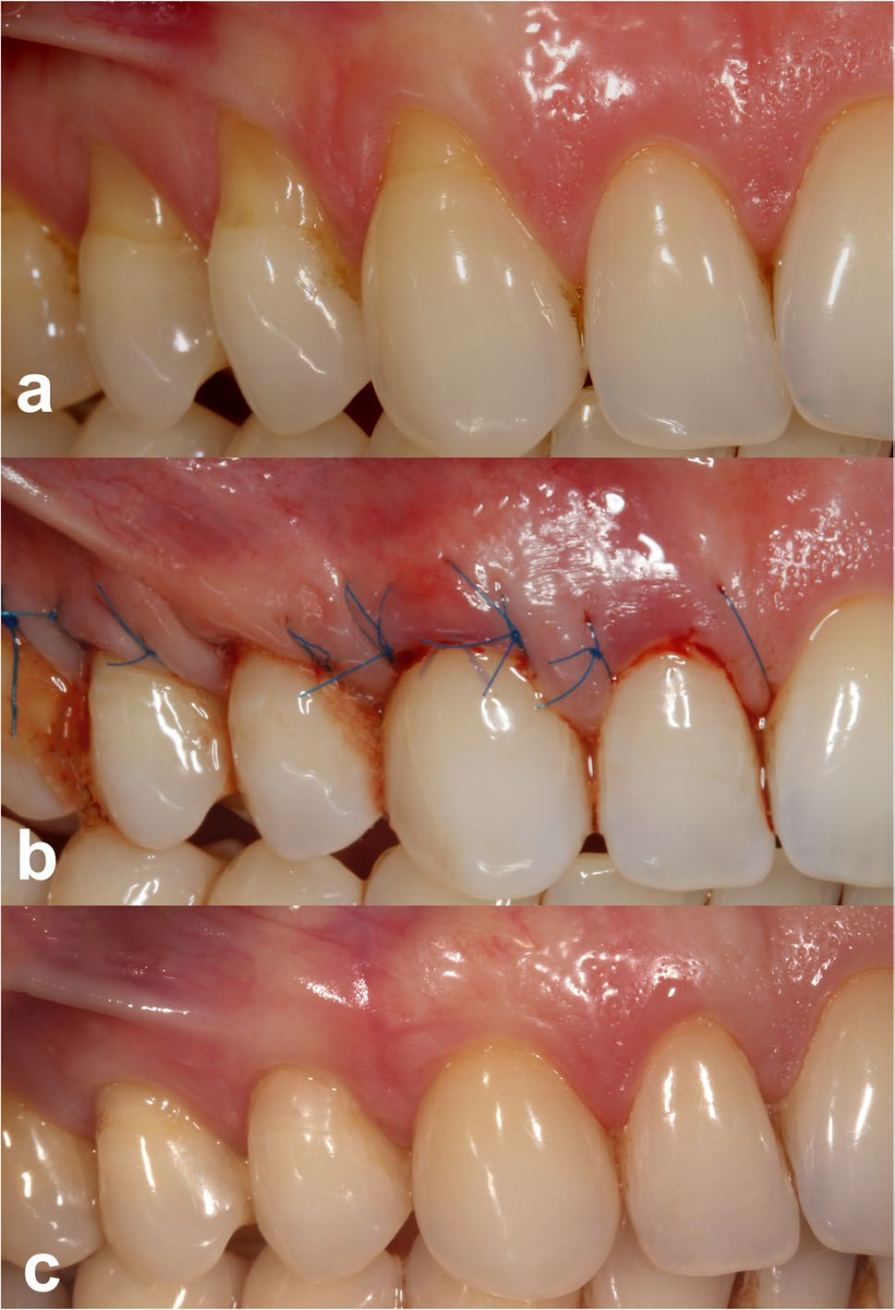
**a** Preoperative view of gingival recessions on control side. **b** Immediate postoperative view. **c** Twelve months postoperative view

Better esthetic results were seen in the test group. The root coverage esthetic score in the SCTG + EMD group was 9.6 ± 0.9, whereas in the SCTG group 8.5 ± 1.1 (p = 0.0429) (Table 3). In addition to average RES, there were also statistically significant differences in four other parameters: marginal tissue contour, soft tissue texture, muco-gingival junction alignment, and gingival color, between the two treatment modalities. All of the abovementioned were in favor of test sites. The gingival margin position did not differ between the treatment modalities. Keloid formation was not observed in any patient after 12 months. Clinical outcomes in one patient are shown in Figs. 2 and 3.

**Table 3:** Evaluation of esthetic outcomes after 12 months (mean and standard deviation)

	GM	MTC	STT	MGJ	GC	RES
SCTG + EMD	5.60 (1.02)	0.99 (0.12)	0.97 (0.17)	0.99 (0.12)	1.00 (0.00)	9.62 (0.93)
SCTG	5.38 (1.22)	0.75 (0.44)	0.78 (0.42)	0.79 (0.41)	0.77 (0.22)	8.51 (1.12)
*p*	0.2291	0.0199*	0.0031*	0.0441*	0.0332*	0.0429*

*SCTG* subepithelial connective tissue graft, *EMD* Emdogain®, *GM* gingival margin, *MTC* marginal tissue contour, *STT* soft tissue texture, *MGJ* muco-gingival junction alignment, *GC* gingival color, *RES* root coverage esthetic score

^*^Statistically significant (*p* ≤ 0.05)

### Logistic regression analysis

In stepwise multivariate analysis, the following parameters were independently associated with 12-month ARC > 85%: EMD application (OR = 7.33; 95%CI = 2.43–12.12), baseline AERSA (OR = 3.56; 95% CI = 1.98–10.19), tooth type (OR = 1.78; 95% CI = 0.45–3.54), and baseline CAL (OR = 0.32; 95% CI = 0.01–2.91) (Table 4).

**Table 4:** Multivariate models based on stepwise logistic regression

Model	Treatment outcome	Predictor	Category or unit	OR [95% CI]	*p*
Model I	ARC	EMD application	No	Reference	
	85%		Yes	7.33 [2.43–12.12]	0.0005
		Tooth type	Incisors/canines/premolars/molars	1.78 [0.45–3.54]	0.0335
		AERSA	1 mm^2^	3.56 [1.98–10.19]	0.0013
		CAL	1 mm	0.32 [0.01–2.91]	0.0432
Model II	CRC	EMD application	No	Reference	
			Yes	21.23 [10.21–45.32]	< 0.0001
		AERSA	1 mm^2^	4.23 [1.11–9.02]	0.0178
		CAL	1 mm	1.29 [0.45–5.81]	0.0329
		GT	1 mm	10.23 [8.37–16.23	< 0.0001
Model III	RES	EMD application	No	Reference	
			Yes	10.23 [5.78–32.23]	< 0.0001
		CAL	1 mm	3.42 [1.87–11.32]	0.0053
		GT	1 mm	5.50 [3.34–16.43]	0.0321
Model IV	KTW gain	Sex	Men	Reference	
			Women	2.75 [1.04–7.79]	0.0474
		Tooth type	Incisors/canines/premolars/molars	0.46 [0.27–0.75]	0.0026
		Tooth position	Upper	Reference	
			Lower	4.09 [1.36–13.86]	0.0164
		PPD	1 mm	1.86 [0.92–3.83]	0.0358
Model V	GT gain	Sex	Men	Reference	
			Women	0.13 [0.03–0.52]	0.0032
		Tooth position	Upper	Reference	
			Lower	4.39 [1.05–15.78]	0.0003
		AERSA	1 mm^2^	5.76 [2.43–12.87]	< 0.0001

*ARC* average root coverage, *EMD* Emdogain®, *AERSA* avascular exposed root surface area, *CAL* clinical attachment level, *CRC* complete root coverage, *GT* gingival thickness, *RES* root coverage esthetic score, *KTW* keratinized tissue width, *PPD* probing pocket depth

For achieving CRC, logistic regression analysis indicated that EMD application, baseline AERSA, CAL, and GT were four significantly associated parameters. Failure to achieve CRC was 21-fold greater in sites that were treated without EMD. The bigger the baseline AERSA and CAL, the less likely 12-month CRC was. With each 1-mm increase in baseline GT, the probability of achieving CRC increased tenfold.

For RES, EMD application, baseline CAL, and GT were significantly associated parameters. EMD application increased tenfold (OR = 10.23; 95% CI = 5.78–32.23) the odds of postoperative RES = 10. The greater baseline CAL, and the smaller baseline GT, the more likely postoperative RES being equal to or lower than 9.

In multivariate analysis, the following parameters independently predicted 12-month KTW gain: sex (OR = 2.75; 95% CI = 1.04–7.79), tooth type (OR = 0.46; 95% CI = 0.27–0.75), tooth position (OR = 4.09; 95% CI = 1.36–13.86); baseline PPD (OR = 1.86; 95% CI = 0.92–3.83).

For GT gain, logistic regression analysis indicated that sex, tooth position, and baseline AERSA remained three statistically significant predictors. The likelihood of failure (postoperative GT ≤ 2 mm) increased for men, upper teeth, and greater values of baseline AERSA.

### Regression model diagnostics

**Fig. 4:**
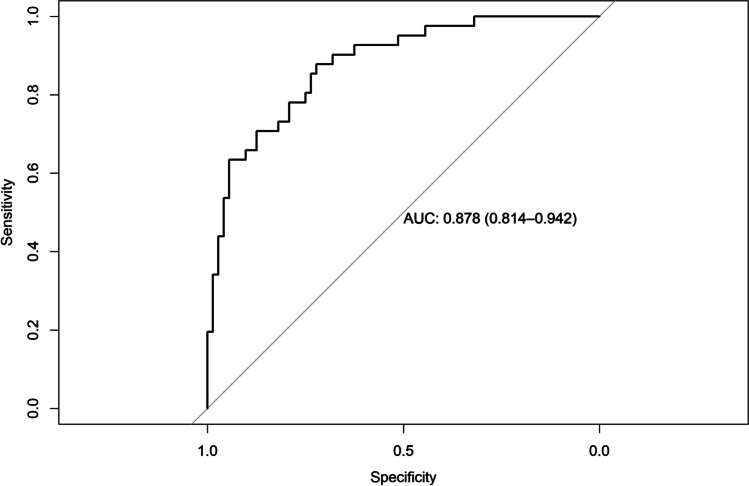
Receiver operating characteristic curve and AUC (0.878; 95% CI 0.81–0.94) for the model predicting ARC

**Fig. 5:**
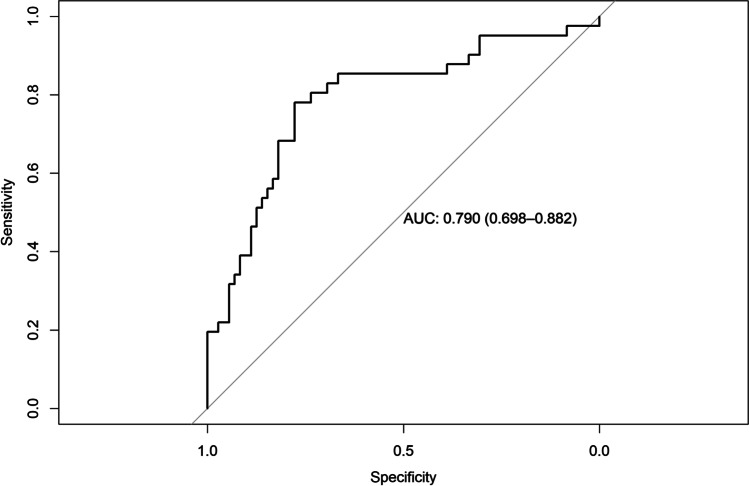
Receiver operating characteristic curve and AUC (0.790; 95% CI 0.69–0.88) for the model predicting CRC

**Fig. 6:**
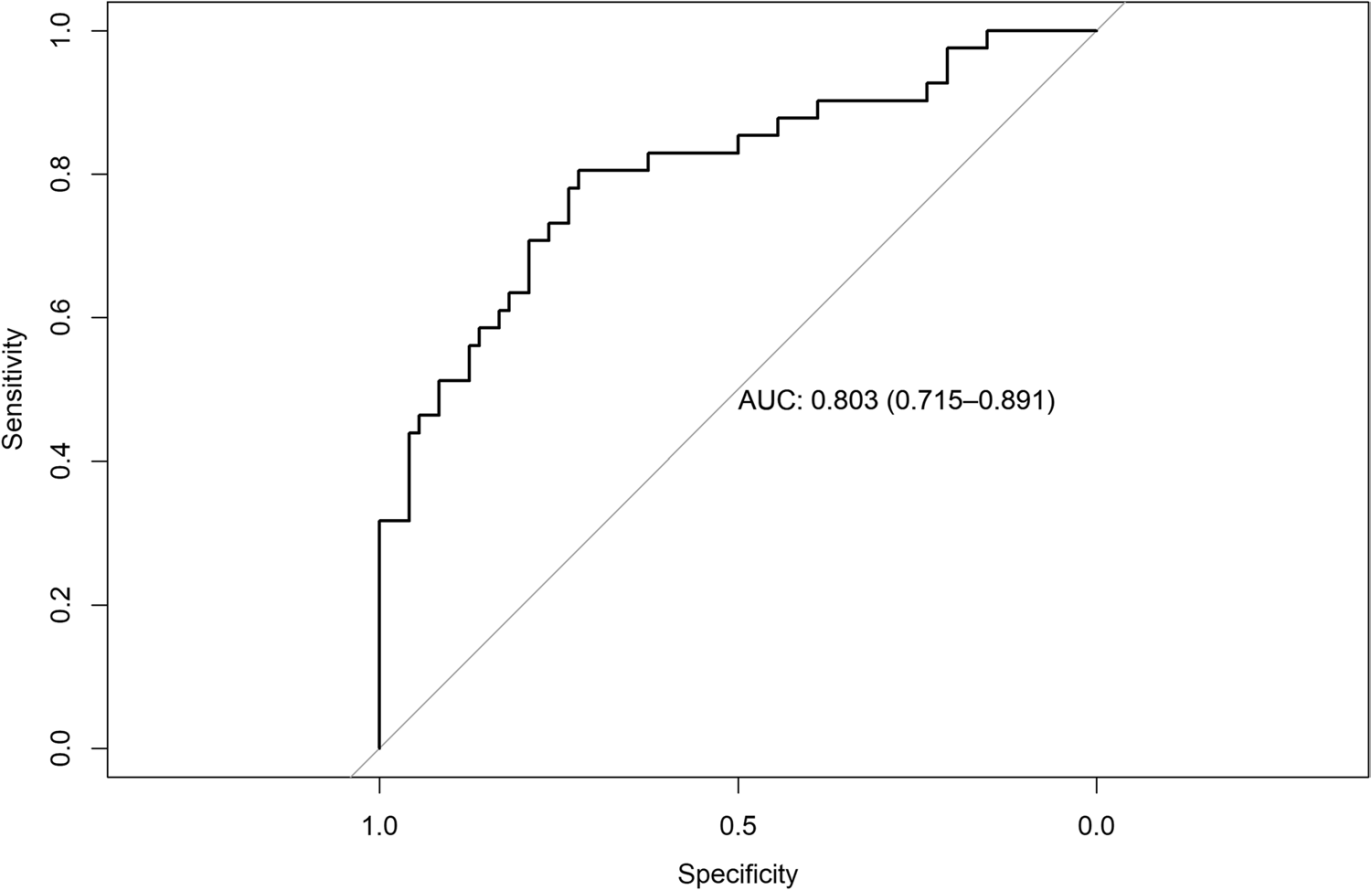
Receiver operating characteristic curve and AUC (0.803; 95% CI 0.71–0.89) for the model predicting RES

**Fig. 7:**
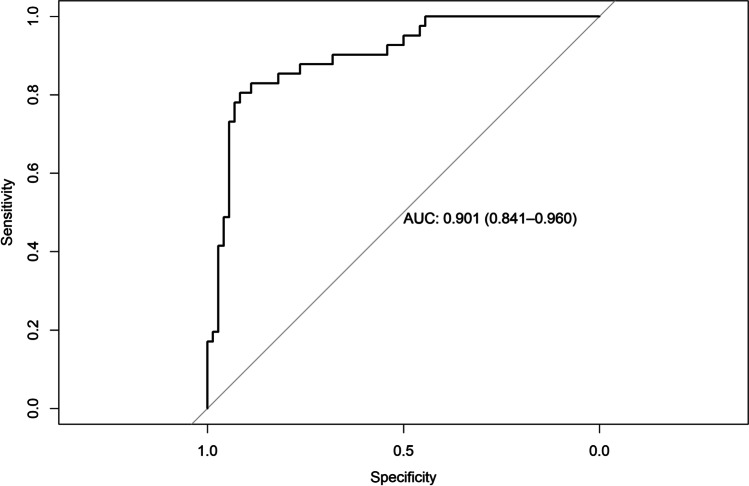
Receiver operating characteristic curve and AUC (0.901; 95% CI 0.84–0.96) for the model predicting KTW gain

**Fig. 8:**
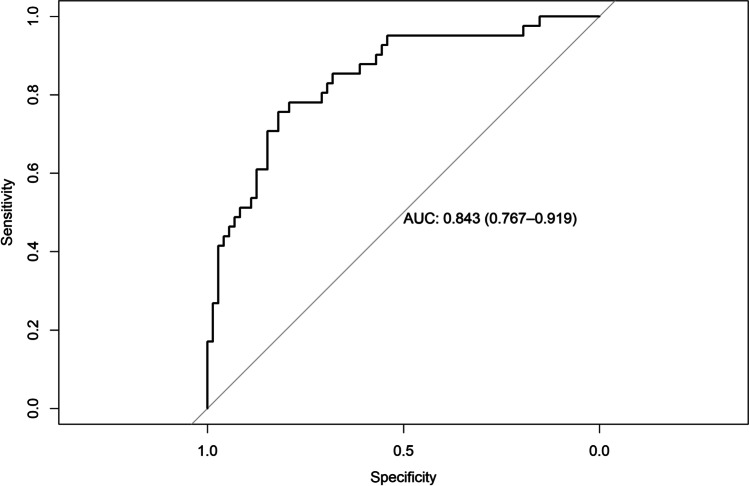
Receiver operating characteristic curve and AUC (0.843; 95% CI 0.76–0.91) for the model predicting GT gain

AUC for the model predicting ARC was 0.878 (95% CI = 0.81–0.94), AUC for the model predicting CRC was 0.790 (95% CI = 0.69–0.88), AUC for the model predicting RES was 0.803 (95% CI = 0.71–0.89), AUC for the model predicting KTW gain was 0.901 (95% CI = 0.84–0.96), and AUC for the model predicting GT gain was 0.843 (95% CI = 0.76–0.91) (Figs. 4, 5, 6, 7, and 8). The Hosmer–Lemeshow goodness of fit (GOF) test associated with the comparison between predicted and observed outcomes did not show statistically significant differences in any evaluated logistic models which confirmed the models being of acceptable fit (Table 5).

**Table 5:** Evaluation of models’ calibration with Hosmer and Lemeshow goodness of fit (GOF) test

	Model I (ARC)	Model II (CRC)	Model III (RES)	Model IV (KTW gain)	Model V (GT gain)
Chi-squared	7.7355	12.2887	7.0493	11.9357	3.2015
Degree of freedom	8	8	8	8	8
*p*	0.4597	0.0945	0.5313	0.1541	0.9211

*ARC* average root coverage, *CRC* complete root coverage, *RES* root coverage esthetic score, *KTW* keratinized tissue width, *GT* gingival thickness

## Discussion

The favorable outcomes of periodontal plastic procedures depend on a plethora of various parameters. In the present study, the influence of selected prognostic factors on average and complete root coverage, root coverage esthetic score, gain in keratinized tissues width, and gain in gingival thickness 12 months after modified coronally advanced tunnel technique was evaluated. To reduce the complexity of statistical analysis, a subset of independent variables was investigated with multiple logistic regression models to assess a measure of the magnitude (OR and 95% CI) of the impact of each variable on the outcomes of interest. To evaluate the applicability of customized models, regression model diagnostics were used. The results indicated that EMD application, baseline AERSA, and baseline GT were the strongest predictors of the clinical outcomes. To the best of our knowledge, this is the first study where such an analysis was carried out in this specific clinical scenario. Although not all initially enrolled patients could be evaluated after 12 months because of some dropouts, the number of subjects remained satisfactory.

MCAT was found to be an extremely effective approach in the treatment of multiple RT1 and RT2, as ARC of 95% for SCTG + EMD-treated and of 91% for SCTG-treated defects were observed. CRC was achieved in 90% (tests) and 86.6% (controls) of the cases. The abovementioned differences were not statistically significant. All in all, the subgroup analysis showed comparable results in terms of GR reduction, as well as KTW and GT gain. However, CRC is not the single purpose of therapy and other factors, such as final soft tissue quality and related esthetics, play a vital role in critical evaluation of clinical success. While 60% of the RES value is attributed to CRC, the remaining 40% is affected by factors such as marginal tissue contour, soft tissue texture, presence of keloid, gingival color, and muco-gingival junction alignment [30]. All of these factors contribute to the esthetics of the smile. Be that as it may, the RES score was significantly better in favor of the SCTG + EMD group (9.62 versus 8.51, p = 0.0429). These findings compare well with the ranges reported by other studies, which utilized similar methodology [5, 10, 13]. Furthermore, TUN + SCTG showed better clinical and volumetric outcomes than CAF + EMD 2 years postoperatively [33]. However, since the adjunctive graft materials were different between treatment arms, an imbalanced comparison was created.

In multivariate logistic regression analysis, the application of EMD was significantly associated with better clinical outcomes. Nevertheless, histological analysis was not performed to evaluate regenerative capacity of EMD; therefore, no comments could be made on the type of tissue formed. The likelihood of ARC > 85% increased sevenfold, of achieving CRC 21-fold, and of gaining perfect RES (10) tenfold in favor of SCTG + EMD-treated defects. Higher overall esthetic performance for root coverage was due to the amount of achieved root coverage, as well as better graft integration with adjacent soft tissues in terms of color and appearance. Consequently, a regression analysis indicated that EMD application offered a clear clinical advantage after root coverage of multiple recessions with MCAT + SCTG technique in this study, even though no differences were observed in the descriptive analyses. It may be explained, at least partially, by several confounding factors affecting simple statistical comparisons between tests and controls. A systematic review that evaluated treatment outcomes when EMD was used in gingival coverage procedures supported the positive effects of EMD determined by enhancing proliferation, attachment, and differentiation of periodontal cells, cementoblasts, and osteoblasts as well as favoring soft tissue regeneration and collagen synthesis and angiogenesis [34]. Very recently, a histological analysis revealed that treatment with CAF + SCTG + EMD resulted in statistically significant shorter epithelium length, greater new cementum, periodontal ligament, and new bone formation, as compared with controls [35]. EMD was found to boost by 20–40% the expression of TFG-β1, TGF-β2, VEGF, IL-1β, MMP-1, versican, and fibronectin in a rat wound healing model [36]. Adjunctive EMD used in root coverage procedures showed clinical advantages for diminishing the duration of postoperative discomfort, pain, and swelling [17, 37]. In a recent meta-analysis, additional application of EMD in treatment of maxillary GR with either CAF or SCTG demonstrated moderate certainty evidence in favor of their use for further reduction in recession height and CAL gain at 6 and 12 months [38]. In addition, the application of EMD affected the stability of gingival margin during the maintenance phase, since the EMD group also showed less recession rebound up to 36 months. The reduction of GR favoring SCTG + EMD was found statistically significant (difference − 0.66 ± 0.48 mm; p = 0.01). Other studies, however, did not show any additional benefits of GR treatment with or without EMD. It was attributed to the technique sensitiveness of EMD application in MCAT technique resulting in some shift in outcomes and discrepancy. Stähli et al. [13] treated 40 patients with Miller class I, II, or III single or multiple GR with MCAT + SCTG with or without EMD and 6 months after surgery they failed to show an influence of EMD on immunological parameters (Il-1β, Il-8, Il-10, MMP-8), clinical outcomes, and patient-reported outcomes. ARC measured 78 ± 26% in the test group and 77 ± 18% in the control group. Moreover, Aroca et al. [18] reported that the use of EMD did not influence root coverage 12 months after treatment of Miller class III multiple gingival recession with MCAT + SCTG, whereas the effects of the distance from the tip of papilla to the contact point (DCP) and the type of tooth were statistically significant. The probability to achieve CRC was more than 89% when DCP at baseline was less than 3 mm for maxillary teeth, while for mandibular teeth, the probability was 34%. The observed disparities may be explained by the fact that in the mentioned studies different types of defects were treated.

Our data were able to highlight the impact of GR dimension as a notable prognostic factor. A multivariate analysis indicated that baseline AERSA remained a statistically significant predictor for ARC, CRC, and GT gain. With each 1-mm^2^ increase in preoperative AERSA, the chance of failure (postoperative ARC ≤ 85%, not achieving 12-month CRC and postoperative GT ≤ 2 mm) increased almost fourfold, fourfold, and almost sixfold, respectively. Based on these results, it is clear that one should expect less root coverage in case of greater baseline AERSA. Nonetheless, single linear measurements (recession height and width) did not emerge as potential prognostic parameters of statistically significant importance in our report. The findings of the present study are not in line with results from a recently published study by Bakhishov et al. [39] who found in multilevel regression analysis that GR height at baseline was a significant negative prognostic factor in the amount of 12-month ARC after MCAT + SCTG (p = 0.02). The mentioned model revealed no significant effect of initial RW on ARC though, which was in accordance with our findings. In another study, greater CRC was noted at sites with baseline recession depth of ≤ 2.5 mm [5]. The detrimental effect of recession dimension on the outcomes achieved after coverage procedures is a consistent finding among numerous clinical trials [20, 24]. Based on our own analysis, the former statement might be further refined and reinforced. Admittedly, AERSA > 15 mm^2^ was reported to have significantly lower odds ratio of having 100% root coverage when compared to AERSA ≤ 15 mm^2^ in a 6-month explorative study on a single GR located at the upper and lower incisors and canines treated with laterally positioned flap [24]. However, because of distinct differences in methodological settings between the mentioned report and the present study, their findings are not directly comparable. In any case, AERSA, being simple multiplication of recession height and width, is only a rough calculation of the exposed area due to root surfaces having irregular dimensions and distinct characteristics. Although conventional linear measurements with a periodontal probe in millimeter scale are reliable, they might also be limited by errors associated with rounded readings and interpretation angles. It is worthy to mention that quite recently, more advanced technologies such as three-dimensional quantitative measurements taken via intraoral scanning were introduced to offer more accurate and precise surgical area assessments [33, 40].

In our study, baseline GT independently predicted 12-month CRC and RES. The greater the baseline GT, the more likely the postoperative chance of achieving CRC and RES = 10. On the other hand, one may speculate that smaller baseline GT might have an impact on poorer multiple root coverage outcome after MCAT + SCTG. A value of 0.8 mm was indicated by some authors to be the critical flap thickness above which CRC might be expected [19]. Stefanini et al. [41] reported that GT < 1 mm influenced the percentage of root coverage. By the same token, the adjunctive use of SCTG in the presence of GT ≤ 1 mm provided high ARC and CRC both in the short and long terms (1–3 years). In another report, Zuhr et al. [33] demonstrated positive correlations between marginal soft tissue thickness (THK) and both ARC and GR reduction. The required minimum mean THK to achieve CRC increased from 1.44 mm (12 months) to 1.61 mm (24 months). It appeared that especially sites with thinner phenotype benefited from minor gingival thickening during surgery, aiming for mean GT of around 1.5 mm. Recently, it has been demonstrated with the help of multilevel model analysis that GT and KTW values at baseline were significant positive prognostic factors of ARC (p = 0.004, p = 0.013, respectively) after MCAT + SCTG [39]. The present results are in partial agreement with these findings, since each increase in baseline GT of 1 mm led to 10 times higher probability of having CRC, but no influence of baseline KTW on 12-month outcomes was observed. Moreover, other authors stated that thickness change 3 mm apical to CEJ yielded inferior results than points located closer to the gingival margin [40]. This information may be of paramount importance for planning surgical treatment. According to some recent studies, SCTG should be added to MCAT in sites presenting a thin phenotype or a reduced band of WKT < 2 mm [15, 42]. Rasperini et al. implemented tunnel approach without additional graft in subjects with thick and very thick phenotype and concluded that the key factor in obtaining root coverage was the amount of KTW, not the use of SCTG itself [42]. It seems also reasonable that addition of SCTG in case of thick phenotype may result in unnatural appearance of the treated site and lower RES [6]. Our study demonstrated that the use of EMD in combination with SCTG added no clinical benefits in terms of KTW gain. These results are in agreement with data presented by other authors and in recent meta-analyses that investigated whether the use of EMD could improve KTW [13, 38, 43]. The abovementioned seemed to be technique dependent. Ribeiro et al. [44] found a KTW increase 3 years after MACT + SCTG leaving the graft exposed. In our study, SCTG was completely submerged to promote revascularization of the graft, and thus, the graft might have behaved differently. All things considered, it is important that apart from root coverage, the treatment goal of GR therapy should be to attain adequately thick and keratinized soft tissue to provide patients with maintainable periodontal conditions for long term.

The current classification of recessions is often used to anticipate the amount of root coverage that can be obtained [21]. In RT1, complete root coverage (CRC) is achievable; for RT2, some studies confirmed the limit of interdental CAL loss within which 100% root coverage is predictable applying different surgical approaches, whereas for RT3 CRC is not possible [18, 21, 23]. Consequently, loss of interproximal CAL alone might not represent a limit in terms of positive outcomes [10]. Aroca et al. [10] obtained predictable, nearly threefold reduction of Miller’s class III multiple recessions 1 year after MCAT in spite of a lack of interproximal bone support. The authors attributed these outcomes to the support of the gingival margin by SCTG, which might in turn have been slightly stretched in the interproximal spaces. This observation is consistent with findings of our study. Having said that, baseline mid-buccal CAL was significantly associated with 12-month ARC, CRC, and RES. The greater baseline mid-buccal CAL, the more likely postoperative failure (ARC ≤ 85%, not achieving CRC, RES ≤ 9). With each 1-mm increase in baseline CAL, the chances of positive outcomes decreased 0.32-fold in terms of ARC (OR = 0.32; 95% CI = 0.01–2.91), 1.29-fold for gaining CRC (OR = 1.29; 95% CI = 0.45–5.81), and with respect to RES (OR = 3.42; 95% CI = 1.87–11.32).

It has previously been demonstrated that tooth position influences ARC and CRC. In fact, maxillary teeth achieved higher root coverage than mandibular teeth [5, 18, 45]. These differences were explained by distinct anatomical characteristics: bigger papillae in the upper arch, and presence of lip muscles and a minor vestibular depth in the lower arch [46]. Moreover, posterior teeth were associated with poorer outcomes compared to anterior teeth [23]. However, location of the tooth should also be assessed with respect to site-specific muco-gingival conditions, such as WKT, GT, and presence of frenuli. In our study, tooth type and tooth position were independently associated with ARC, KTW gain, and GT gain. A logistic regression analysis indicated that the probability of postoperative ARC > 85% and postoperative KTW > 3 mm increased in case of premolars, when compared to incisors (OR = 1.78; 95% CI = 0.45–3.54 and OR = 0.46; 95% CI 0.27–0.75, respectively). Moreover, for lower teeth the chance of 12-month KTW greater than 3 mm and GT greater than 2 mm was 4-times higher (OR = 4.09; 95%CI = 1.36–13.86; and OR = 4.39; 95%CI = 1.05–15.78, respectively). The results of this study suggest greater KTW and GT gain in favor of premolars, and lower teeth, which are quite interesting findings. In the light of this, the results of the present study will contribute to the literature on the subject.

On the assumption that individualized predictions are utilized for clinical decision-making, well-evaluated estimates are paramount. With knowledge of this, regression model diagnostics is applied to assess how accurately models describe the underlying relationships between predictors and outcomes of interest [47]. In our study, the validity of logistic regression models was evaluated through discrimination and calibration, both of which are readily understandable by the medical community. Discrimination refers to the ability of a model to correctly ascribe a higher risk of an outcome to the subjects who are precisely at higher risk, whereas calibration refers to the ability of the model to ascribe precise average absolute level of risk (accurate probability of an event occurring). Designed models predicting 12-month ARC > 85%, CRC, RES = 10, and GT gain had fair discrimination, whereas a model predicting KTW gain had excellent discrimination. In the ROC analysis, the area under ROC curves showed high values, which meant that the models correctly identified defects with stronger likelihood of achieving postoperative outcomes of interest. In order to accurately estimate the probability of a specific outcome, the next step was to assess calibration. The Hosmer–Lemeshow statistic showed that all models were well calibrated, which meant that the overall magnitude of likelihood was predicted accurately, while residuals were minimized. Taking into account the overall evaluation, all the models depicted in our study were accurate. We tried to present findings of this report in accordance to available data in the published literature. Since these results could not be directly compared to previous studies, further investigations with similar methodology are necessary to test the robustness of the presented models. The question how different results could be explained by other variables or distinct subjects’ characteristics must remain open at this point of time. It should be kept in mind, that up-to-the-point prediction of future root coverage is sometimes not feasible, even if all the factors of clinical importance are scrutinized.

Some limitations have to be taken into consideration while evaluating findings of our study. Firstly, when multiple variables are closely related to one another in logistic regression, collinearity may occur. As a result, errors in predictions of effects of these variables on designated outcomes might happen. Moreover, in case of one predictor altering the value of another predictor, a possibility of interaction needs to be considered. It is advised to work with a reasonable number of predictive variables in order to decrease systematic errors that stemmed from data gathering using too many variables. Secondly, there are some inherent limitations of logistic regression diagnostics. Assessing values of AUC, apart from extreme values, might be subject to interpretation, whereas the Hosmer–Lemeshow statistics rely on the number of risk groups. In small samples, the Hosmer–Lemeshow test has low power and may fail to recognize poorly calibrated models. In some scenarios, more complex statistical metrics should be applied, although some sophisticated analyses might produce algorithms much too difficult to be used clinically. Despite these limitations, the present study relied on well-accepted methods of regression model diagnostics. Thirdly, logistic regression models in our study were built with specific justifiable end points for clinical outcomes of interest. All of them discriminated well and had good calibration at these designated points; however, shifted cut points would be associated with different sensitivities and specificities. What is more, the population of patients in this study was relatively young, and the estimates of the models for other age groups should be regarded with caution. In case of application regression models to population that differs from population they were built on, large residuals will be difficult to account for. Therefore, it is crucial to understand how a model will be employed in practice. Given the potential selection bias, further research is needed for external validation, determination of accuracy, and evaluation of clinical utility of the depicted models. Lastly, different variables such as flap tension, dimension of interdental papillae, operator’s skill, and the center effect should be examined to enhance prognostic models for multiple root coverage in future studies on a more representative case mix.

Within these limitations, this is the first study that evaluated probability of specific 12-month clinical outcomes after root coverage of multiple GR with MCAT + SCTG + EMD using baseline clinical characteristics of treated defects in logistic regression approach. The split-mouth design was adapted to minimize individual differences depending on patient, when compared with research designed as a parallel group. Ultimately, the reported findings should provide clinicians with insights into medical decision-making and prognosis of MCAT for multiple RT1 and RT2.

## Conclusions

Considering the limitation of the present study, it can be concluded that:

- Additional use of EMD to MCAT + SCTG in soft tissue augmentation of multiple RT1 and RT2 provides higher OR of achieving ARC > 85%, CRC, and RES = 10, 12 months postoperatively.
- The greater the baseline dimension of GR (measured as avascular exposed root surface area), the greater likelihood of failure 12 months after MCAT + SCTG (ARC ≤ 85%, not achieving CRC, postoperative GT < 2 mm).
- Baseline GT determines higher OR for achieving 12-month CRC and perfect RES (10) after treatment of multiple recessions with MCAT + SCTG.
- Tooth type and tooth position might influence postoperative ARC, KTW gain, and GT gain.
- All the designed predictive models exhibit fair to excellent discrimination and satisfactory calibration in patients aged 20–40 years old.
- The presented models may help clinicians develop individualized treatment for patients presented with multiple RT1 and RT2.
